# Natural killer cell antibody‐dependent cellular cytotoxicity to *Plasmodium falciparum* is impacted by cellular phenotypes, erythrocyte polymorphisms, parasite diversity and intensity of transmission

**DOI:** 10.1002/cti2.70005

**Published:** 2024-11-01

**Authors:** Stephen Tukwasibwe, Savannah Nicole Lewis, Yoweri Taremwa, Kattria van der Ploeg, Kathleen D Press, Maureen Ty, Felistas Namirimu Nankya, Kenneth Musinguzi, Evelyn Nansubuga, Florian Bach, Martin Chamai, Martin Okitwi, Gerald Tumusiime, Annettee Nakimuli, Francesco Colucci, Moses R Kamya, Joaniter I Nankabirwa, Emmanuel Arinaitwe, Bryan Greenhouse, Grant Dorsey, Philip J Rosenthal, Isaac Ssewanyana, Prasanna Jagannathan

**Affiliations:** ^1^ Infectious Diseases Research Collaboration Kampala Uganda; ^2^ School of Medicine, Uganda Christian University Mukono Uganda; ^3^ Department of Medicine Stanford University Stanford CA USA; ^4^ School of Medicine, Makerere University Kampala Uganda; ^5^ Department of Obstetrics and Gynaecology University of Cambridge Cambridge UK; ^6^ Department of Medicine University of California San Francisco San Francisco CA USA

**Keywords:** antibody‐dependent cellular cytotoxicity, malaria, natural killer cells

## Abstract

**Objectives:**

Natural killer (NK) cells make important contributions to anti‐malarial immunity through antibody‐dependent cellular cytotoxicity (ADCC), but the role of different components of this pathway in promoting NK cell activation remains unclear.

**Methods:**

We compared the functions and phenotypes of NK cells from malaria‐exposed and malaria‐naive donors, and then varied the erythrocyte genetic background, *Plasmodium falciparum* strain and opsonising plasma used in ADCC to observe their impacts on NK cell degranulation as measured by CD107a mobilisation.

**Results:**

Natural killer cells from malaria‐exposed adult Ugandan donors had enhanced ADCC, but an impaired pro‐inflammatory response to cytokine stimulation, compared to NK cells obtained from malaria‐naive adult North American donors. Cellular phenotypes from malaria‐exposed donors reflected this specialisation for ADCC, with a compartment‐wide downregulation of the Fc receptor γ‐chain and enrichment of highly differentiated CD56^dim^ and CD56^neg^ populations. NK cell degranulation was enhanced in response to opsonised *P. falciparum* schizonts cultured in sickle cell heterozygous erythrocytes relative to wild‐type erythrocytes, and when using opsonising plasma collected from donors living in a high transmission area compared to a lower transmission area despite similar levels of 3D7 schizont‐specific IgG levels. However, degranulation was lowered in response to opsonised field isolate *P. falciparum* schizonts isolated from clinical malaria infections, compared to the 3D7 laboratory strain typically used in these assays.

**Conclusion:**

This work highlights important host and parasite factors that contribute to ADCC efficacy that should be considered in the design of ADCC assays.

## Graphical abstract

Natural killer (NK) cells make important contributions to anti‐malarial immunity through antibody‐dependent cellular cytotoxicity (ADCC), but the role of different components of this pathway in promoting NK cell activation remains unclear. We compared the functions and phenotypes of NK cells from malaria‐exposed and malaria‐naive donors, and then varied the erythrocyte genetic background, *Plasmodium falciparum* strain, and opsonising plasma used in ADCC to observe their impacts on NK cell degranulation as measured by CD107a mobilisation. Here, we show how NK cell donor, *Pf* origin, intensity of malaria transmission and erythrocyte genetic background can differentially modulate the activation of NK cells during ADCC.

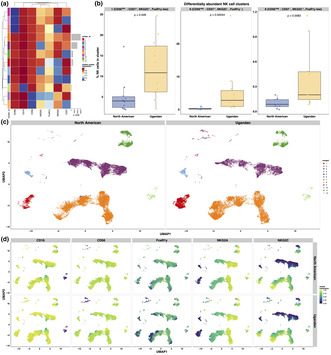

## Introduction

Malaria, a vector‐borne disease caused by *Plasmodium falciparum* (*Pf*), remains a major challenge to global health. In 2022, there were an estimated 249 million cases of malaria resulting in 608 000 deaths, 76% of which occurred in children under the age of 5 years.^1^ The burden of malaria is highest in sub‐Saharan Africa, which consistently accounts for approximately 95% of global cases and deaths.^1^ Naturally acquired immunity to malaria is non‐sterilising and exposure‐dependent, culminating in protection from symptoms (anti‐disease immunity) and reduced parasite biomass (anti‐parasite immunity) following infection.^2^ Individuals living in endemic areas become protected from severe malaria early in life, but repeated exposure is required to gain protection from mild symptoms, resulting in the asymptomatic infections common in older children and adults.^3^, ^4^ Mechanisms driving this clinical protection are multifactorial. Some amount of inflammatory signalling is necessary to activate immune cells to control parasitaemia; however, overproduction of inflammatory cytokines during malaria infection is strongly associated with severe disease and fatal outcomes. Therefore, tight regulation of parasite‐induced inflammation is likely required to allow asymptomatic infection.^5^

Clinical immunity to malaria is also reliant on the development of malaria‐specific antibodies,^6^, ^7^, ^8^ with increasing evidence that Fc‐dependent effector mechanisms play a critical role in mediating anti‐parasite protection.^9^, ^10^, ^11^ NK cells, a versatile type of innate lymphoid cell present in peripheral blood, can secrete inflammatory cytokines that activate and enhance the effector activities of other cell types^12^ and can also eliminate *Pf*‐infected erythrocytes (iRBCs) directly through antibody‐dependent cellular cytotoxicity (ADCC).^10^, ^11^ The NK cell population as a whole is heterogeneous, and cells are further classified into subpopulations on the basis of CD56 and CD16 expression. The CD56^dim^ CD16^+^ subset comprises a majority of the peripheral blood population in immunocompetent adults, and is capable of mediating ADCC, natural cytotoxicity in response to major histocompatibility complex I (MHC‐I)‐deficient targets and secreting pro‐inflammatory cytokines.^12^, ^13^ However, a population of CD56^dim^ NK cells with low expression of the signalling Fc receptor gamma chain (FcεR1γ) identified in cohorts of malaria‐exposed Malian children and adults was shown to have enhanced ADCC activity against opsonised iRBCs and was associated with resistance to symptomatic infection.^14^, ^15^ We recently found that Ugandan children repeatedly exposed to malaria had elevated frequencies of CD56^neg^ CD16^+^ NK cells, and that this population had enhanced ADCC activity relative to CD56^dim^ CD16^+^ cells.^16^ The presence of this population was also correlated with protection from symptomatic malaria, but the frequency of CD56^neg^ CD16^+^ NK cells declined rapidly in the setting of interrupted transmission.^16^

Together, these data suggest that NK cell activity may be modified by repeated exposure to malaria parasites, similar to what has been described in the context of human cytomegalovirus (CMV) infection.^17^, ^18^, ^19^ However, the extent to which malaria exposure modifies the NK cell compartment of individuals living in malaria endemic regions remains unclear. In addition, although ADCC mediated by NK cells has emerged as a highly relevant facet of anti‐malarial immunity,^16^, ^20^, ^21^ to our knowledge, no studies have evaluated the separate components of this process to observe their impact on NK cell activity. To address these gaps, we analysed NK cells from Ugandan and North American adult donors to identify cellular features associated with endemic malaria exposure. We also analysed whether the degree of malaria transmission, erythrocyte genetic background, or the origin of *Pf* strains influenced the ability of NK cells to perform ADCC against iRBCs.

## Results

### NK cells from malaria‐exposed Ugandan adults are superior to those from North Americans at mediating ADCC, but have an impaired inflammatory IFNγ response

The effector capabilities of immune cells can vary between donors because of a variety of factors, including genetic characteristics and past exposure to pathogens. Studies of cell‐mediated antibody effector responses to *Pf* typically leverage NK cell samples from malaria‐naive or exposed donors without direct comparison, leaving the donor's influence on experimental results unclear. To address this uncertainty, we used several *in vitro* functional assays to identify differences in NK cell effector functions between endemically exposed and malaria‐naive NK cell donors.

We analysed circulating NK cell populations from peripheral blood mononuclear cells (PBMCs) collected in 2022 from adult donors in Eastern Uganda where malaria transmission is holoendemic and year‐round^22^ (Supplementary table 1) and malaria‐naive North American donors. Live CD3^−^, CD14^−^, CD19^−^, CD7^+^ NK cells were further classified into subsets based on CD56 and CD16 expression as CD56^bri^ (CD56^+^, CD16^−^), CD56^dim^ (CD56^+^, CD16^+^) and CD56^neg^ (CD56^−^, CD16^+^) (Supplementary figure 1a). We first assessed the capacity of NK cells from both donor groups to perform ADCC in response to purified schizont‐infected red blood cells (iRBCs) from the 3D7 laboratory strain opsonised with pooled plasma from malaria‐naive North Americans or immune Ugandans. We used the marker CD107a (LAMP‐1), which becomes detectable when cytotoxic intracellular granules fuse to the cell membrane during degranulation,^23^ to quantify NK cell activation by flow cytometry. CD107a expression on NK cells has also been correlated with specific lysis of iRBCs in prior work.^11^

**Figure 1:**
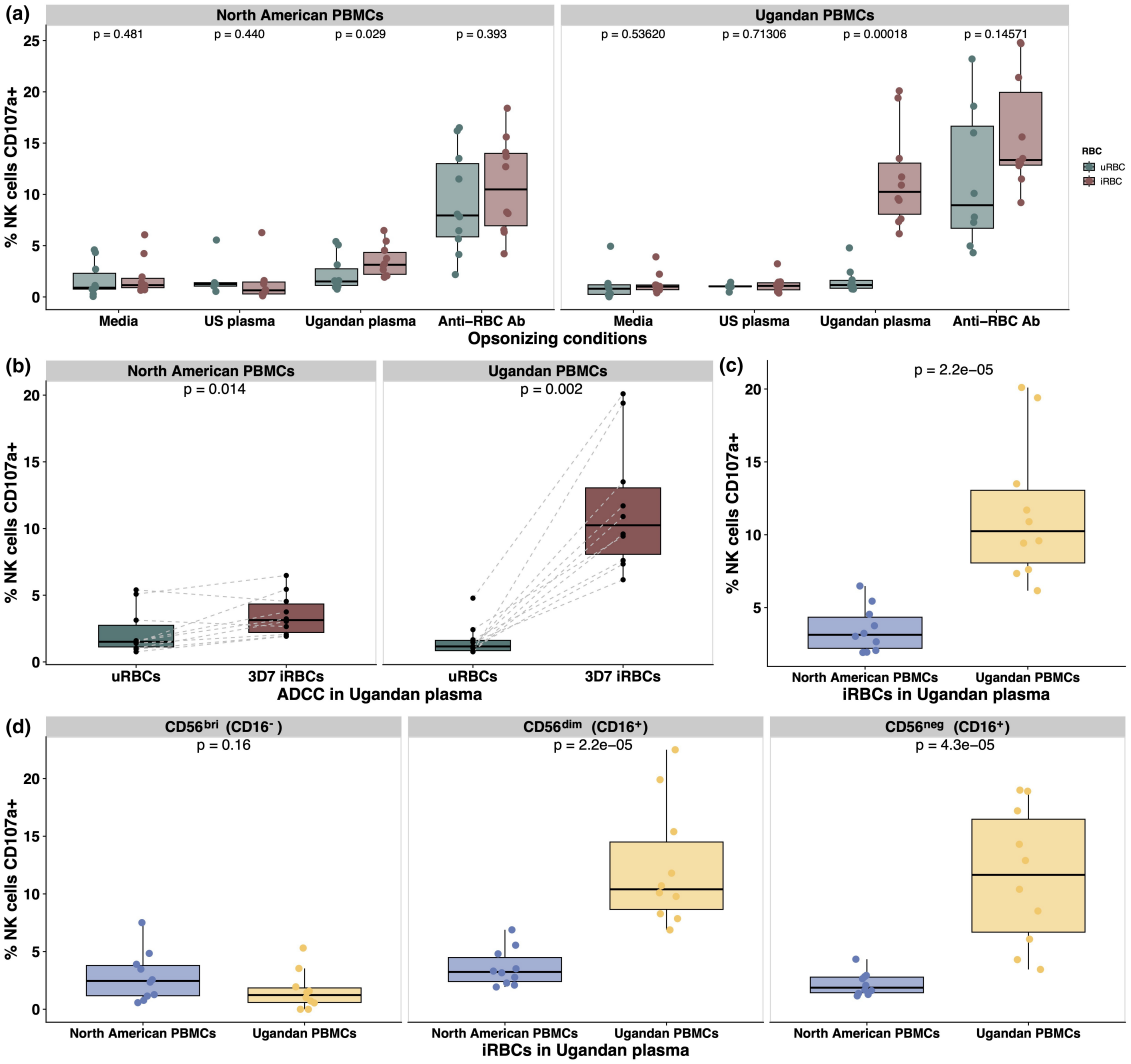
NK cells from malaria‐exposed Ugandan adults had ADCC activity superior to that of malaria‐naive North Americans. NK cell degranulation was quantified using the marker CD107a (LAMP‐1). PBMCs from North American (*n* = 10) and Ugandan (*n* = 10) donors were stimulated with iRBCs and uRBCs either alone or opsonised with serum at a ratio of 2 iRBCs to 1 PBMC **(a)**. Comparison of North American and Ugandan NK cell degranulation in response to iRBCs opsonised in pooled Ugandan serum in all NK cells **(b, c)** and in individual NK cell subpopulations **(d)**. *P‐*values were calculated using Wilcoxon rank sum tests (paired in **b** only). ADCC, antibody‐dependent cellular cytotoxicity; iRBCs, infected erythrocytes; NK, natural killer; PBMCs, peripheral blood mononuclear cells.

Although NK cells from both donor groups degranulated in the presence of *Pf* 3D7 iRBCs opsonised with pooled Ugandan plasma (Figure 1a and b), NK cells from Ugandan donors degranulated significantly more than those from North Americans (*P*‐value = 2.2e‐5) (Figure 1c). This difference was driven by the CD56^dim^ (*P*‐value = 2.2e‐5) and CD56^neg^ (*P*‐value = 4.3e‐5) NK cell subpopulations, which are capable of mediating ADCC on the basis of CD16 expression (Figure 1d). NK cells from both groups did not degranulate in response to unopsonised iRBCs, iRBCs opsonised with plasma from malaria‐naive donors or uninfected red blood cells (uRBCs) opsonised in pooled Ugandan plasma, indicating that the observed responses were because of the presence of *Pf*‐specific antibodies (Figure 1a). There were no significant differences in degranulation when PBMCs were incubated with RBCs opsonised in a polyclonal anti‐RBC antibody (Supplementary figure 1b). To test whether these findings were due to differences in CD16 downstream signalling or specific to opsonised iRBCs, we performed a CD16 crosslinking experiment as previously described^24^ to measure NK cell effector responses to plate‐bound anti‐CD16 antibodies. We observed no significant differences in CD107a expression or IFNγ production between NK cells from North American and Ugandan donors (Supplementary figure 1c, d).

**Figure 2:**
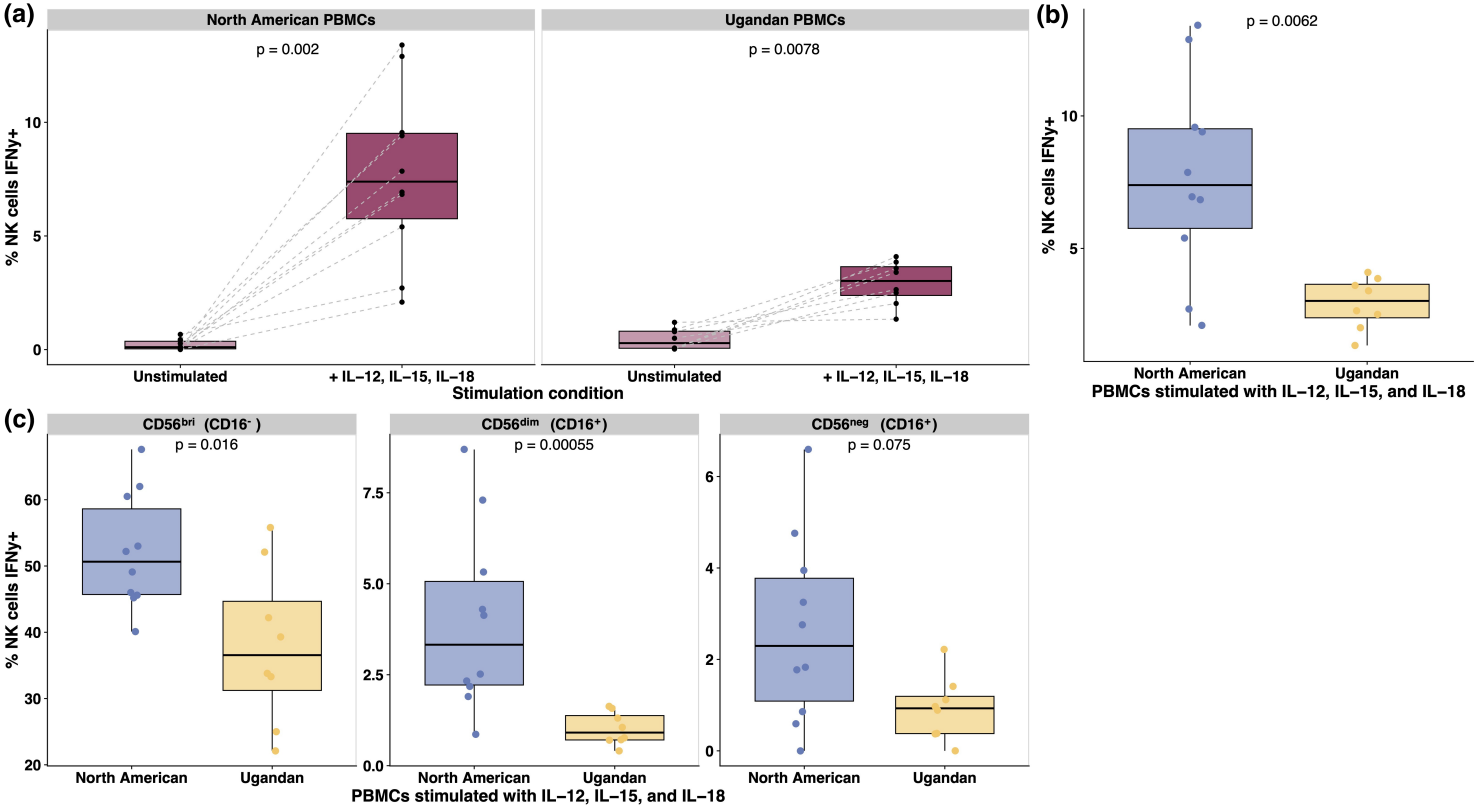
NK cells from malaria‐exposed donors had a poor inflammatory response to cytokine stimulation. PBMCs from North American (*n* = 10) and Ugandan (*n* = 8) donors were stimulated with a cocktail of IL‐12 (2.5 ng mL^−1^), IL‐15 (10 ng mL^−1^) and IL‐18 (0.25 μg mL^−1^) or medium. NK cell activation was quantified by staining for intracellular IFNγ. Comparison of North American and Ugandan NK cell IFNγ production following stimulation in all NK cells **(a, b)** and within NK cell subpopulations **(c)**. *P‐*values were calculated using Wilcoxon rank sum tests (paired in **a** only). NK, natural killer; PBMCs, peripheral blood mononuclear cells.

Next, we stimulated PBMCs from both donor groups with a cocktail of monocyte‐derived cytokines IL‐12, IL‐15 and IL‐18 to investigate pro‐inflammatory cytokine production by activated NK cells.^12^, ^25^, ^26^ We found that NK cells from North American donors produced significantly more IFNγ in response to cytokine stimulation than Ugandan NK cells (*P*‐value = 0.0062) (Figure 2a and b). The majority of IFNγ produced in both donor groups was from the CD56^bri^ subset, consistent with the canonical role of these cells as pro‐inflammatory mediators with little to no cytotoxic activity^26^ (Figure 2c) IFNγ production was significantly attenuated in Ugandan CD56^bri^ (*P*‐value = 0.016) and CD56^dim^ (*P*‐value = 0.00055) NK cell populations in comparison to the same subsets from North American donors (Figure 2c).

Together, these results showed that NK cells from malaria‐exposed Ugandan adults activated strongly in response to opsonised iRBCs, but were relatively poor at responding to and producing pro‐inflammatory cytokines. Differences in degranulation were specific to iRBCs opsonised in pooled Ugandan plasma, as there was no significant difference in the ability of North American and Ugandan NK cells to degranulate in response to RBCs opsonised in a polyclonal anti‐RBC antibody (Supplementary figure 1b) or to plate‐bound anti‐CD16 antibodies (Supplementary figure 1c, d). In contrast, NK cells from malaria‐naive North Americans were able to mount a significantly more robust IFNγ response than Ugandan NK cells when stimulated by cytokines, but were relatively worse at performing ADCC.

### NK cells from Ugandan adults were phenotypically mature and highly differentiated with adaptive features

We hypothesised that the functional specialisation of Ugandan NK cells to perform ADCC would correspond to differential expression of activating and inhibitory markers between malaria‐exposed and malaria‐naive donors. To test this, we performed an extensive comparative analysis of NK cell phenotypes from adult Ugandan and North American PBMC donors. Using flow cytometry, we measured expression of the extracellular markers CD57, CD85j (LILRB1), NKG2A, NKG2C, NKp46, NKp80 and multiple killer‐cell immunoglobulin‐like receptors (KIRs), as well as the intracellular Fc adaptor protein, FcRγ (FcεR1γ). We used unsupervised clustering and uniform manifold approximation and projection (UMAP) to analyse and visualise these data.^27^

**Figure 3:**
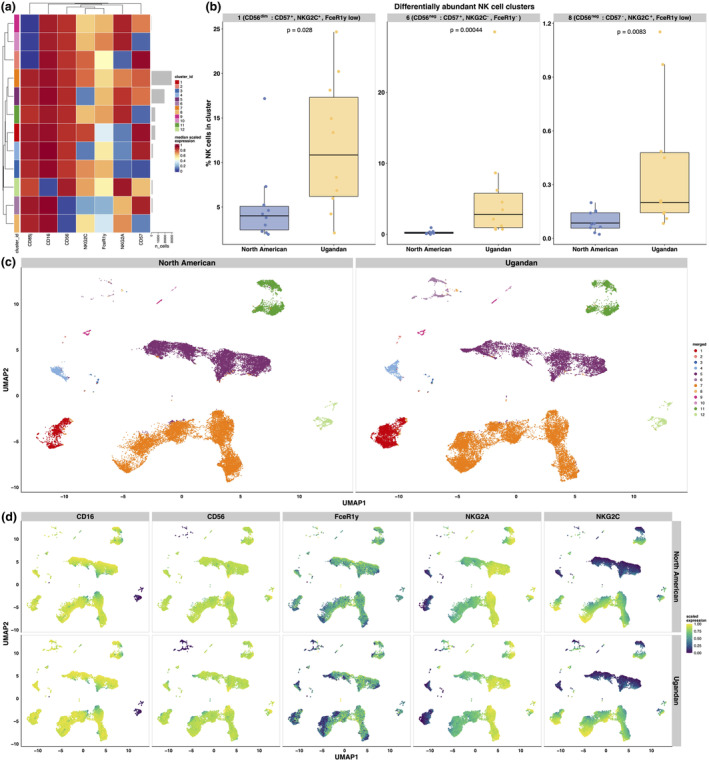
Endemic malaria exposure was associated with phenotypic maturity and extensive differentiation in NK cells. **(a)** Heat map showing the phenotypic characteristics of defined NK cell clusters identified from North American (*n* = 10) and Ugandan PBMC donors (*n* = 12). Marker expression was scaled between 0 and 1 by using lower and upper quantiles as boundaries prior to aggregating the data. Row and column clustering were performed using unscaled data. **(b)** Bar graphs representing the individual NK cell clusters that were found to be significantly enriched in Ugandan PBMC donors through differential abundance testing within CATALYST. *P‐*values were calculated using unpaired Wilcoxon rank sum tests. **(c)** UMAP visualisation of NK cells from North American (left) and Ugandan (right) donors, coloured by their corresponding cluster. **(d)** UMAP visualisations of NK cells from North American (top row) and Ugandan Expression data were scaled between 0 and 1 using lower and upper expression quantiles. NK, natural killer; PBMCs, peripheral blood mononuclear cells.

The majority of NK cells identified were conventional CD56^dim^ populations with varying expression of the markers NKG2A and NKG2C (Figure 3a). After creating UMAP visualisations of these clusters, which were stratified by donor group, we observed that two specialised populations of NK cells appeared to be more abundant in Ugandan donors: FcRγ^−^ CD56^dim^ and CD56^neg^ FcRγ^−^ populations (Figure 3c, Supplementary figure 2a). We confirmed through differential cluster abundance analysis that these populations were indeed significantly enriched in Ugandan donors compared to North Americans (Figure 3b). Additionally, we observed that expression of FcRγ was markedly reduced across the entire NK cell compartment of Ugandan PBMC donors (Figure 3d).

This observation of reduced FcRγ expression in Ugandan NK cells was consistent with our manual gating data: all NK cells from Ugandan donors had significantly reduced expression of not only FcRγ (*P*‐value = 0.0011), but also NKG2A (*P*‐value = 0.0071) and NKp80 (*P*‐value = 0.023) compared to cells from North American donors (Supplementary figure 2c). We also observed that relative to cells from North Americans, all NK cells from Ugandans had significantly increased expression of CD85j (*P*‐value = 0.021) (Supplementary figure 2c). Although nonsignificant, CD57 (*P*‐value = 0.059) and NKG2C (*P*‐value = 0.11) expression was also elevated in NK cells from Ugandan donors relative to North Americans, with the latter potentially because of higher regional seroprevalence of CMV^16^ (Supplementary figure 2c). KIR expression was similar between donor groups (Supplementary figure 2c). When we filtered these data to examine only CD56^dim^ and CD56^neg^ cells, we found that these manual gating data were generally in agreement with our unsupervised clustering. (Supplementary figure 2d).

Together, these data show that the NK cell compartment of malaria‐exposed Ugandan adults had a phenotypic profile consistent with a high degree of maturity and differentiation. We found that phenotypically specialised CD56^dim^ and CD56^neg^ subpopulations were significantly enriched in Ugandan donors, and were consistent with previous descriptions of adaptive and adaptive‐like NK cells in malaria‐exposed individuals.^14^, ^15^, ^16^, ^17^, ^18^, ^19^, ^25^ Importantly, these characteristics were not shared by NK cells from malaria‐naive North American donors.

### NK cell degranulation was reduced in response to erythrocytes infected with clinical isolates of *Pf*

Our functional assays revealed that NK cells from Ugandan PBMC donors degranulate significantly more than those from North American donors in response to 3D7 *Pf* iRBCs opsonised in pooled Ugandan plasma. Because different *Pf* strains can be distinct and can influence the protective efficacy of adaptive immune responses,^28^, ^29^ we next investigated whether iRBCs isolated from locally acquired malaria infections differentially influence NK cell degranulation during ADCC.

3D7 and clinical isolate iRBCs obtained from Tororo, Uganda were cultured in parallel and schizonts were purified at similar percent parasitaemia. We opsonised both 3D7 and clinical isolate iRBCs using pooled Ugandan plasma, and then incubated them with NK cells purified from Ugandan adult PBMC donors. We again used the marker CD107a to quantify NK cell activation by flow cytometry. We found that NK cell degranulation was significantly higher in the presence of opsonised 3D7 *Pf* in comparison to clinical isolate *Pf* (*P‐*value = 0.0061*)* (Supplementary figure 3). Degranulation beyond background levels did not occur when either 3D7 or clinical isolate iRBCs were opsonised in opsonin‐free medium, indicating that malaria‐specific antibodies in the pooled Ugandan plasma mediated this effect. There was also no significant difference in degranulation in the positive control condition, in which iRBCs were opsonised with a polyclonal anti‐RBC antibody (Supplementary figure 3).

These data show that NK cells degranulated less following incubation with clinical isolate iRBCs opsonised in pooled Ugandan plasma in comparison to opsonised 3D7 iRBCs, but the precise mechanisms underlying these results remain unclear. Although neither total nor antigen‐specific antibody binding of *Pf* isolates was quantified, the lack of differences observed when opsonising 3D7 vs. clinical isolates with an anti‐RBC antibody suggests that ADCC differences may represent unequal antibody binding of the plasma pools to these isolates.

### High‐transmission donor plasma is a more potent activator of NK cell degranulation than low‐transmission plasma

Individuals living in high malaria transmission settings have higher levels of malaria‐specific avid antibodies than individuals living in lower transmission settings,^30^, ^31^, ^32^ but whether transmission intensity also impacts malaria‐specific ADCC has been unknown. We sourced plasma from children living in three locations with varied malaria transmission: Walakuba, Jinja District, a peri‐urban area with relatively low transmission intensity (annual entomological inoculation rate [aEIR] of 3.8 infectious bites per person per year [PPY]); Kihihi, Kanungu District, a rural area in Western Uganda with moderate transmission intensity (aEIR of 32 infectious bites PPY); and Tororo, Tororo District, a rural area in south‐Eastern Uganda with high transmission intensity (EIR of 301 infections bites PPY; Supplementary table 2).^33^ IgG titres to 3D7 schizont lysate as measured by an enzyme‐linked immunosorbent assay (ELISA) were similar in the three plasma pools (Supplementary figure 4a).

**Figure 4:**
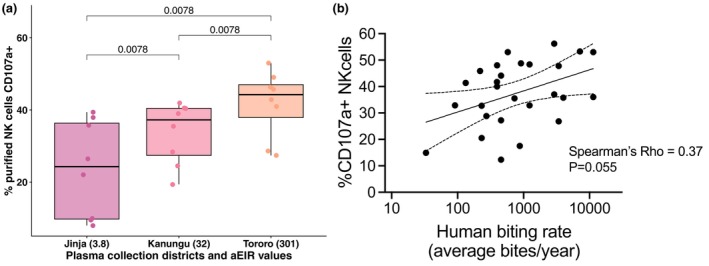
The intensity of malaria transmission at the site of opsonising plasma collection was positively correlated with NK cell degranulation during ADCC. Opsonising plasma was obtained from children living in three areas of Uganda with different local intensities of malaria transmission. **(a)** NK cells purified from Ugandan PBMC donors (*n* = 3, three independent experiments) were stimulated with iRBCs opsonised in pooled plasma from Jinja (aEIR = 3.8), Kanungu (aEIR = 32) and Tororo (aEIR = 301). *P‐*values were calculated using paired Wilcoxon rank sum tests. **(b)** Correlation of NK cell degranulation in response to iRBCs incubated in plasma from a subset of the individual donors used to create pools in **a** with their corresponding household‐level human biting rate values. For this scatterplot, Rho (ρ) and *P*‐values were calculated using Spearman's correlation. ADCC, antibody‐dependent cellular cytotoxicity; iRBCs, infected erythrocytes; NK, natural killer; PBMCs, peripheral blood mononuclear cells.

Purified NK cells degranulated significantly more in response to iRBCs incubated in pooled high transmission Tororo plasma in comparison to iRBCs incubated in low transmission Jinja plasma (*P*‐value = 0.0078) (Figure 4a). This trend remained consistent when we further compared degranulation between 3D7 and clinical isolate iRBCs (Supplementary figure 4b). Moreover, we observed a positive correlation between NK cell degranulation in response to iRBCs incubated in plasma from individual donors used to create pools in Figure 4a with corresponding household‐level human biting rate (Spearman's rho = 0.37, *P*‐value = 0.055) (Figure 4b). Altogether, these data demonstrate that when donor plasma is used to opsonise iRBCs, the local intensity of malaria transmission correlated positively with NK cell degranulation during ADCC.

### Sickle cell trait‐carrying erythrocytes were associated with enhanced NK cell degranulation during ADCC

Sickle cell trait (HbAS genotype) is thought to promote resistance to malaria infection through physical, biochemical and immune mechanisms.^34^ Accumulating evidence suggests that the magnitude of protection afforded by HbAS increases with age,^35^, ^36^, ^37^ suggesting the involvement of adaptive immune responses. However, the precise ways in which this genotype influences adaptive effector activity remain unclear. Since iRBCs are typically derived from cultures in wild‐type HbAA erythrocytes, we investigated whether 3D7 *Pf* cultured in sickle cell heterozygous (HbAS) erythrocytes would impact the magnitude of NK cell degranulation during ADCC.

**Figure 5:**
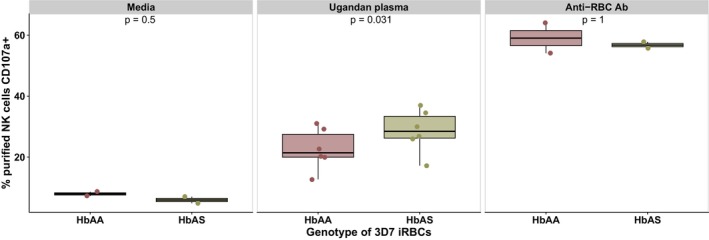
NK cell degranulation in response to opsonised iRBCs was enhanced by sickle cell trait. iRBCs were obtained from *Pf* cultures supplemented with blood with an HbAA or HbAS background. NK cells purified from Ugandan PBMC donors (*n* = 2, two independent experiments) were stimulated with iRBCs alone or opsonised in serum. *P‐*values were calculated using paired Wilcoxon rank sum tests. iRBCs, infected erythrocytes; NK, natural killer; PBMCs, peripheral blood mononuclear cells.

3D7 iRBCs cultured in HbAA or HbAS red blood cells were cultured in parallel and purified at similar parasitaemia. Schizont stage iRBCs from each culture were opsonised in pooled Ugandan plasma, and were then used to stimulate NK cells purified from adult Ugandan PBMC donors. In comparison to exposure to HbAA iRBCs, degranulation was significantly higher when NK cells were exposed to HbAS iRBCs (*P*‐value = 0.031) (Figure 5). No degranulation occurred in the opsonin‐free medium condition, and there was no significant difference in degranulation in the positive control anti‐RBC antibody condition. These data suggest that red blood cell polymorphisms such as HbAS may impact the ability of NK cells to perform ADCC, although the precise mechanisms remain unresolved.

## Discussion

Natural killer cell activity has been associated with protection from symptomatic malaria across several endemic settings and patient demographics,^15^, ^16^, ^21^ suggesting that ADCC mediated by NK cells may be one of several essential determinants of clinical immunity. Therefore, it is crucially important to evaluate this process in laboratory settings that best mirror the complexity of *in vivo* biological conditions. In this work, we considered a number of factors involved in ADCC that might influence the magnitude of NK cell degranulation.

We first discovered that the choice of donor source of NK cells used in ADCC assays is a critically important factor to consider. We compared the functional and phenotypic characteristics of NK cells from both malaria‐exposed and malaria‐naive adult PBMC donors. Following exposure to 3D7 iRBCs opsonised in pooled Ugandan plasma, NK cells from malaria‐exposed Ugandans demonstrated a significantly increased capacity to mediate ADCC in comparison to NK cells from malaria‐naive North Americans (Figure 1). Importantly, we also found that differences in ADCC were specific to iRBCs opsonised in Ugandan plasma, as there was no difference in the ability of North American or Ugandan NK cells to respond to RBCs opsonised in a polyclonal anti‐RBC antibody (Supplementary figure 1b) or activate in response to CD16 stimulation (Supplementary figure 1c, d). Our immunophenotyping and unsupervised clustering analysis of Ugandan and North American NK cells revealed that several clusters of FcRγ^−^ CD57^+^ CD56^dim^ and CD56^neg^ cells were significantly enriched in Ugandan samples (Figure 2). NK cells lacking expression of FcRγ have been associated with enhanced ADCC activity across multiple infectious and noninfectious disease states.^38^, ^39^, ^40^, ^41^ In the context of malaria, FcRγ^−^ CD56^dim^ NK cells demonstrated potent ADCC activity in several endemic settings^14^, ^42^ and have been identified as protective from symptomatic malaria in a cohort of Malian adults.^15^ We speculate that differences in the ability to respond to iRBCs opsonised by malaria‐specific antibodies are driven by the presence of specialised, highly differentiated NK cell subpopulations with an intrinsically higher capacity to produce an effector response to immune complexes. Given the lack of significant differences between Ugandan and North American NK cells to anti‐RBC ADCC conditions and CD16 crosslinking, we speculate that additional host–parasite interactions^43^, ^44^, ^45^, ^46^, ^47^ may provide additional activating signals to NK cells in addition to those conferred by the Fc domains of malaria‐specific antibodies.

Although NK cells lacking expression of CD56 have been described as exhausted or dysfunctional in different infectious disease contexts,^48^, ^49^ we recently discovered a highly functional population of FcRγ^−^ CD56^neg^ NK cells in Ugandan children with an ADCC phenotype that was strongly correlated with protection from symptomatic malaria.^16^ A bulk RNA‐seq study^50^ of NK cells from malaria‐exposed Kenyan children with Burkitt's lymphoma showed that CD56^neg^ NK cells have not only reduced expression of transcripts encoding FcRγ, but also reduced expression of transcripts encoding NKp46, NKp80 and CD85j. This transcriptional profile is consistent with cellular surface expression we observed in Ugandan adults, and may suggest that NK cells in malaria‐exposed populations may become functionally specialised over time to optimally perform ADCC. Longitudinal sampling of individuals from endemic regions paired with lineage tracing of clonal NK cell populations^51^ could conclusively prove this hypothesis.

We also observed that when we stimulated the same set of donor PBMCs with the monocyte‐derived cytokines IL‐12, IL‐15 and IL‐18 and measured NK cell activation through IFNγ production,^12^, ^25^ NK cells from Ugandan PBMC donors had a significantly impaired inflammatory response when compared to NK cells from North American donors (Figure 3). Malaria is a highly inflammatory infection, and many studies have shown that pro‐inflammatory cytokines and their associated downstream signalling pathways are highly correlated with severe malaria outcomes and poor patient outcomes.^5^, ^52^, ^53^ Individuals who become clinically immune to malaria experience asymptomatic infections.^4^, ^54^ Our results support a model in which inflammatory NK cell responses are dampened to prevent pathological immune signalling by activated cells, while their antibody‐dependent cytotoxic activity is simultaneously expanded to aid in controlling parasitemia.^24^ This may be achieved through epigenetic mechanisms, as is described in NKG2C^+^ adaptive NK cells with enhanced effector functions in the context of CMV infection.^17^, ^18^, ^19^, ^55^ Indeed, previous work from our group described extensive and distinct differences in histone modifications between CD56^neg^ NK cells with enhanced ADCC activity in malaria‐exposed Ugandan children and typical CD56^dim^ cells.^16^

In addition to describing donor‐dependent differences in NK cell functions and phenotypes, we observed that the origin of *Pf* in iRBCs has an influence on ADCC. When compared to the 3D7 laboratory strain, the magnitude of NK cell degranulation was significantly reduced when cells were incubated with opsonised clinical isolate iRBCs (Supplementary figure 3). In both natural settings and controlled human malaria infections, the risk of infection and disease severity varies depending on the parasite strain used, which suggests the genetic background of the parasite may influence the subsequent immune response.^28^, ^29^, ^56^, ^57^ Several NK cell receptors have been reported to interact with host and parasite‐derived ligands expressed on the surface of iRBCs,^43^, ^44^, ^45^, ^46^, ^47^ though the ultimate immunological consequences of these interactions remain to be determined.^47^ Genetic and proteomic characterisation of the clinical isolates used were not performed herein, and thus differences relative to the 3D7 strain and specific mechanisms driving our observation remain unclear. It is known that *Plasmodium var* genes, which encode highly diverse surface‐expressed proteins that mediate cytoadherence and drive antigenic variation, are epigenetically regulated^58^ and expression is greatly impacted by adaptation to *in vitro* culture.^59^, ^60^, ^61^, ^62^, ^63^ While it is possible that some antigenic characteristics in the clinical isolate were preserved, it is likely that *var* gene expression was profoundly affected by culture. As the degree of antibody binding to the *Pf* isolates used in the Supplementary figure 3 experiments was not quantified, further work is needed to identify antigens expressed by both clinical isolate and 3D7 *Pf*, and whether differential expression of antigens bound by antibodies in donor plasma alone is sufficient to explain differences in ADCC outcomes.

We also investigated how local malaria transmission affects NK cell degranulation during ADCC by creating pools of opsonising plasma from Ugandan children living in areas with differing intensity of malaria transmission. We observed that opsonising plasma from high transmission areas was more stimulatory to NK cells than plasma from lower transmission areas despite having similar titres of 3D7‐specific IgG (Figure 4, Supplementary figure 4a). No difference was found in malaria‐specific IgG titres in a larger serological study of paediatric samples collected at a similar time interval in Uganda^30^; however, this same study described an inverse relationship between transmission intensity and avidity that has been documented in another highly endemic setting.^64^ Although we did not profile antigen‐specific antibody responses in this analysis, prior work has shown that IgG to both merozoite (Rh5, AMA‐1 and MSPs) and erythrocyte stage (PfEMP1) antigens is associated with exposure in some settings^30^, ^31^, ^64^ and protection in others.^65^, ^66^, ^67^, ^68^, ^69^ We did not profile IgG subclasses in these plasma pools, but protective malaria‐specific antibodies are typically IgG3 and IgG1, and both have a similar capacity to engage with Fc receptors.^67^, ^68^, ^70^ It is also possible that differential antibody glycosylation in endemic areas could lead to increased NK cell activation by changing binding affinity to CD16.^71^ Indeed, a recent report described that afucosylation of malaria‐specific PfEMP1 increased with parity in a cohort of pregnant Ghanaian women and with age in Ghanaian children and potently activated NK cells during ADCC.^20^, ^72^ Precise links between the local intensity of malaria transmission, antibody glycosylation and cellular effector responses have yet to be drawn, especially with broader study cohorts of malaria‐exposed individuals.

Lastly, we observed that the magnitude of NK cell degranulation during ADCC increased when opsonised 3D7 iRBCs were cultured in HbAS erythrocytes compared to results with HbAA erythrocytes (Figure 5). Sickle cell trait is speculated to promote resistance to symptomatic malaria through both biochemical and immune mechanisms, and may accelerate the development of clinical immunity to malaria in endemic settings by promoting the generation of adaptive immune responses.^32^, ^33^, ^34^, ^35^ It is also possible that sickle cell trait disrupts parasite physiology and antigen expression, as prior work demonstrated that HbAS iRBCs cytoadhere to epithelial cells worse relative to HbAA iRBCs potentially because of the disrupted and aberrant PfEMP1 expression.^73^ The physiological characteristics of HbAS iRBCs may enhance their vulnerability to NK cell‐mediated cytotoxicity, and possibly other effector responses, which subsequently facilitates the dissemination and presentation of *Pf* antigens to adaptive immune cells. Altered PfEMP1 expression may also facilitate binding of malaria‐specific antibodies to iRBCs, and therefore promote ADCC, in addition to other protective Fc‐driven effector responses. However, the precise mechanism resulting in greater NK cell degranulation in response to opsonised HbAS iRBCs compared to opsonised HbAA iRBCs remains unclear in our experimental conditions.

There were limitations to this study. We intentionally compared NK cells from malaria‐exposed Ugandan adults against those from malaria‐naive North Americans to evaluate the most common experimental setups for ADCC assays in the literature; further exploration into the NK cell biology of Ugandan adults from higher and lower transmission areas is warranted. Background activation in ADCC experiments was consistently low, but it is possible that some nonspecific binding to RBCs occurred: this can be addressed by either ABO‐matching opsonising plasma and blood used in *Pf* culture, or by culturing *Pf* in O‐ blood. Although the functional and phenotypic differences we identified between Ugandan and North American PBMC donors were prominent, our functional characterisation of NK cells from both donor groups was limited to ADCC because of the cell count limitations, so finer aspects of region‐specific differences in NK cell biology remain to be explored. Although we validated our flow cytometry gating strategy in previous work,^16^ the ontology of the rare NK cell populations in our phenotypic analysis, including CD56^neg^ cells, remains unclear, and it is possible that some of these populations may be better classified as an innate lymphoid cell subset.^74^ None of the adults in our study cohort were infected with HIV, but virtually all tested positive for CMV.^16^ Participants were not screened for Epstein–Barr virus (EBV), but prior work in a similar East African setting reported a relatively high seroprevalence of EBV early in life.^75^ Impacts of CMV and EBV infections on our studied responses are unknown. We did not screen plasma pools used in this work for reactivity to specific *Pf* antigens or glycosylation status, which leaves potential mechanistic explanations for our findings regarding *Pf* strains and the intensity of transmission unresolved. It is also possible that our characterisation of regional malaria‐specific antibody titres is not representative of said regions because of methodological and sampling limitations. Epidemiological associations with clinical immunity as they relate to NK cell effector functions were also outside the scope of this work.

As evidence that multifunctional antibodies are strongly associated with protective immunity to malaria continues to surface,^76^, ^77^ it is becoming increasingly evident that Fc receptor‐dependent effector activities are crucial in mediating this effect. ADCC mediated by NK cells in response to iRBCs has recently emerged as a key predictor of protection from symptomatic infection across multiple studies.^15^, ^16^, ^20^, ^21^ In this work, we show how the NK cell donor, *Pf* origin, intensity of malaria transmission and erythrocyte genetic background, can differentially modulate the activation of NK cells during ADCC. By identifying several parameters that influence ADCC efficacy that future investigators can factor into the design of ADCC assays, this work could enhance the translational potential of immunology studies in malaria‐endemic settings.

## Methods

### Study participants and samples

Peripheral blood mononuclear cells were obtained from anonymous adult blood donors from the Stanford Blood Center (California, USA), anonymous adult blood donors from Mbale Regional Referral Hospital (Mbale district, eastern Uganda), or adults enrolled in the East African International Center of Excellence in Malaria Research (ICEMR) PRISM border cohorts in Tororo and Busia district, eastern Uganda^22^ (Supplementary table 1). Malaria transmission in eastern Uganda is holoendemic and perennial, with two seasonal peaks. Plasma samples were obtained from children enrolled in the ICEMR PRISM 1 cohorts from three locations with varying degrees of malaria transmission: Walakuba, Jinja District, a peri‐urban area with relatively low transmission intensity (aEIR of 3.8 infectious bites per PPY); Kihihi, Kanungu District, a rural area in Western Uganda with moderate transmission intensity (aEIR of 32 infectious bites PPY); and Tororo, Tororo District, a rural area in south‐Eastern Uganda with high transmission intensity (EIR of 301 infections bites PPY, Supplementary table 2).^31^ *Plasmodium falciparum‐*infected red blood cells were collected from ICEMR cohort study participants with confirmed malaria by microscopy for use in assays with clinical isolate iRBCs. Anonymous blood donors for parasite culture were recruited from Tororo District General Hospital, with sickle cell trait status confirmed by Gazelle Hb Variant point‐of‐care testing (Hemex Health, Portland, Oregon, USA). Written informed consent was obtained from all participants, including the parent or guardian of all paediatric participants. The study protocols were approved by the Uganda National Council of Science and Technology (HS 1019 and HS 2700), the Makerere University School of Medicine Research and Ethics Committee (2011–167 and 2019‐134), the University of California, San Francisco Committee on Human Research (11–05995 and 19‐28606) and the Institutional Review Boards at Stanford University (41197).

### PBMCs and plasma isolation

For both Ugandan and North American blood bank specimens, PBMCs were isolated from whole blood or white blood cell fractions by density gradient centrifugation (Ficoll‐Histopaque; GE Life Sciences), counted and cryopreserved in liquid nitrogen. Analysis of cell viability using Guava ViaCount (MilliporeSigma, Burlington, Massachusetts, USA) or Countess Automated Cell Counter (Thermo Fisher Scientific, Waltham, Massachusetts, USA) for experiments performed in Uganda or the United States, respectively, consistently demonstrated > 90% viability after thaw. Plasma was obtained from whole blood among individuals enrolled in ICEMR cohorts at the time of routine monthly blood draws. Both individual and plasma pools from individuals from regions with varied malaria transmission intensities or from de‐identified plasma from North American donors were used in the ADCC assay (described below).

### NK cell isolation

Natural killer cells were negatively isolated from freshly thawed PBMCs using the Human NK Cell Isolation Kit (Miltenyi Biotec, Bergisch Gladbach, NRW, Germany) for experiments performed in Uganda. First, cells were resuspended with a chilled buffer (1× PBS with 0.5% BSA and 0.5 m EDTA) and mixed with NK cell Biotin‐Antibody cocktail for 5 min at 4°C. Then, the provided NK Cell Microbead cocktail was added and incubated for 10 min at 4°C. This was followed by magnetic cell separation using an LS column (Miltenyi Biotec). Flow through containing enriched NK cells was collected, washed and left to rest overnight at 37°C in R10 (RPMI 1610 [MilliporeSigma] with 10% FBS, 1% L‐Glutamine [Thermo Fisher Scientific], 1% Penicillin‐ streptomycin [Corning Inc, Corning, New York, USA]) medium supplemented with 400 IU mL^−1^ IL‐2.

### Functional assays

#### Parasite culture and isolation

*Plasmodium falciparum* 3D7 asexual stage and clinical isolates were cultured in RPMI 1640 supplemented with 25 mm HEPES, 25 mm sodium bicarbonate, 1% gentamycin, and enriched with 0.5% Albumax (pH 6.75) and 250 μm hypoxanthine. Parasitaemia was measured every other day by counting Giemsa (MilliporeSigma) stained blood smears, and cultures were maintained at less than 10% parasitaemia. Culture flasks were kept at 37°C under atmospheric conditions (3% oxygen, 5% carbon dioxide and 92% nitrogen), and cultures were routinely enriched with donor red blood cells with either HbAA or HbAS phenotypes to maintain 2% haematocrit. To retain synchronous cultures, cultures were treated with 5% D‐sorbitol for 15 min at 37°C. D‐sorbitol was washed off with incomplete RPMI (RPMI 1640 with 25 mm HEPES, 25 mm sodium bicarbonate, 1% gentamycin), and the culture was re‐incubated for 28 h to reach schizont stage. Schizont‐stage infected cells were isolated using MACS cell separation LD columns (Miltenyi Biotec) after determining that cultures were free of *Mycoplasma* (MycoAlert Mycoplasma Detection Kit, Lonza [Basel, Basel‐Stadt, Switzerland]). After aliquoting purified schizonts, 25 μL of FBS and an equal volume of glycerolyte were added to the aliquots before freezing at −80°C. Frozen aliquots were stored in liquid nitrogen. For experiments, schizonts were thawed and washed using 3.5% NaCl, 1.8% NaCl and PBS, and then resuspended in R10 before addition to cells. The schizonts were counted using a haemocytometer.

#### Antibody‐dependent cellular cytotoxicity (ADCC) assay

Freshly thawed schizont‐stage iRBCs were incubated in incomplete RPMI medium and either 10% plasma (pooled US/naive, pooled immune Ugandan plasma or autologous plasma) or polyclonal anti‐red blood cell antibody (rabbit anti‐human, ab34858, Abcam, Cambridge, UK) at a 1:100 concentration. The iRBCs were incubated for 1 h at 37°C. Following opsonisation, cells were washed with incomplete RPMI, resuspended in R10 medium, and were then added to 96‐well plates containing either PBMCs or purified NK cells for experiments performed in the United States or Uganda, respectively, at a 1:2 effector to target ratio. Instrumentation available for experiments performed in Uganda necessitated the use of purified NK cells for ADCC experiments, but we have shown in prior work^16^ that results of ADCC assays using PBMCs and purified NK cells are comparable. CD107a (BioLegend [San Diego, California, USA] clone H4A3, Brilliant Violet 711) and the ER inhibitors GolgiStop (containing monensin, BD Biosciences [Becton, Dickinson and Company, Franklin Lakes, New Jersey, USA]) and GolgiPlug (containing brefeldin A, BD Biosciences) were immediately added to each well. The 96‐well plate was then centrifuged (100 *g*, 3 min) before incubating for 5 h at 37°C.

Following incubation, the plate was centrifuged (300 *g*, 5 min), washed once with 1× PBS, then incubated with surface antibodies at room temperature for 30 min. The surface antibody master mix included LIVE/DEAD Fixable Aqua Stain (Invitrogen [Thermo Fisher Scientific]), CD16 (BioLegend clone 3G8, Brilliant Violet 650), CD56 (BioLegend clone HCD56, Brilliant Violet 605), CD14 (BioLegend clone M5E2, Brilliant Violet 510), CD19 (BioLegend clone H1B19, Brilliant Violet 510), CD3 (BioLegend clone SK7, APC H7), CD7 (BioLegend clone CD7‐B67, Alexa Fluor 700) and 1xPBS (gating strategy shown in Supplementary figure 1a). After 30 min, FIX & PERM Medium A (Thermo Fisher Scientific) was added and left to incubate for 10 min. Cells were then washed twice with FACS buffer (1× PBS with 0.5% BSA and 0.5 m EDTA) before intracellular staining. The intracellular antibody IFNy (BioLegend clone 4S.B3, Brilliant Violet 785) was added to FIX & PERM Medium B (Thermo Fisher Scientific), and left to incubate for 20 min in the dark at room temperature. After 20 min, cells were washed twice with FACS buffer (1× PBS with 0.5% BSA and 0.5 m EDTA), then resuspended in 1× PBS before flow cytometry. Data were collected on a BD Accuri flow cytometer (4 colours) or an Attune Nxt flow cytometer (12 colours) for experiments performed in Uganda or the United States, respectively. Data were analysed using FlowJo X software (Tree Star).

#### NK cell phenotyping

Thawed PBMCs were rested at 37°C for 1 h before staining. Cells were then washed with 1xPBS and incubated with the following surface antibodies, in addition to the gating antibodies described above, at room temperature for 30 min: CD85j (Thermo Fisher Scientific clone HP‐F1, eFluor 450), KIR2DL1/DS1 (Miltenyi Biotec clone 11 PB6, FITC), KIR3DL1 (BioLegend clone DX9, Brilliant Violet 711), KIR2DL2/L3 (BioLegend clone DX27, PE), NKG2A (Miltenyi Biotec clone REA110, PE), NKG2C (Miltenyi Biotec clone REA205, APC), CD57 (BioLegend clone HNK‐1, PerCP Cy5.5), NKp46 (BioLegend clone 9E2, Brilliant Violet 605), NKp80 (BioLegend clone 5D12, APC). After 30 min, cells were incubated in FIX & PERM Medium A (Thermo Fisher Scientific) for 10 min at room temperature. Cells were washed twice with FACS buffer (1× PBS with 0.5% BSA and 0.5 m EDTA). The intracellular antibody FcRγ (MilliporeSigma clone FCABS400F, FITC) was resuspended in FIX & PERM Medium B (Thermo Fisher Scientific), and left to incubate for 20 min in the dark at room temperature. Cells were then washed twice with FACS buffer (1× PBS with 0.5% BSA and 0.5 m EDTA), then resuspended with 1× PBS before collection on the Attune NXT flow cytometer. Data were analysed using FlowJo X software (Becton Dickinson).

#### Unsupervised phenotypic analysis

Raw data (.fcs) files for live CD3^−^, CD14^−^, CD19^−^ and CD7^+^ cells (gating strategy shown in Supplementary figure 1a) were exported out of FlowJo for further analysis of NK cells. These files were down‐sampled using a function that randomly selects cells within a user‐defined threshold and aspects of flowCore^78^ to reduce oversampling bias in samples with higher cell counts. After preprocessing, the R package CATALYST^27^ was used to perform k‐means clustering and create UMAP visualisations of clustered NK cell populations. Identification of clusters that were significantly enriched between sample groups was also performed within CATALYST^27^ using the package diffcyt^79^: briefly, cluster frequencies were arcsine‐square‐root transformed before normalisation, and clusters with *P*‐values under 0.05 were considered significantly enriched.

#### Cytokine stimulation

After resting PBMCs for 1 h at 37°C, a cocktail containing IL‐12 (2.5 ng mL^−1^, R&D Systems [Bio‐Techne, Minneapolis, Minnesota, USA]), IL‐15 (10 ng mL^−1^, PeproTech [Thermo Fisher Scientific]) and IL‐18 (0.25 μg mL^−1^, R&D Systems) suspended in R10 medium was added to cells. Cells were then incubated for a total of 5 h at 37°C. GolgiPlug (containing brefeldin A, BD Biosciences) was added after 1 h of incubation. After 5 h, cells were stained and collected on the Attune NXT flow cytometer.

#### Enzyme‐linked immunosorbent assay (ELISA)

Schizont lysate was thawed at room temperature and pulsed briefly to remove precipitants. Lysate was diluted 1:4000 in 1× PBS. A volume of 50 μL diluted lysate was added to each well of an ELISA plate, sealed, and incubated overnight at 4°C. The following day, treated were washed three times with PBS‐T (1× PBS supplemented with 0.05% TWEEN 20 [Sigma‐Aldrich, MilliporeSigma]) before incubating in 1% casein blocking buffer (1× PBS supplemented with casein from bovine milk [Sigma‐Aldrich, MilliporeSigma]) for 2 h at 37°C. Plasma samples were diluted in 0.1% casein antibody buffer (50 mL 1% casein blocking buffer added to 500 mL 1× PBS), then 50 μL of diluted plasma were added to treated wells and incubated for 1 h at room temperature. Wells were then washed three times with PBS‐T before incubating 50 μL of secondary antibody (goat anti‐human IgG Fc‐HRP, SouthernBiotech [Birmingham, Alabama, USA]) diluted 1:50 000 in 0.1% casein blocking buffer for 1 h. with three times PBS‐T, then 50 μL 1‐Step TMB substrate (34029, ThermoFisher Scientific) was added to each well and incubated for 15 min at room temperature underneath aluminium foil. 1 m sulphuric acid solution was then added to stop the reaction, and optical density (OD) was quantified at 450 nm using a SpectraMax iD3 multi‐mode microplate reader (Molecular Devices LLC, San Jose, California, USA).

### Statistical analysis

All statistical analyses were performed using STATA version 16 (College Station), SPICE v.5.3 (NIAID) or R version 4.2.0. Comparisons of cellular percentages between groups were performed using the Wilcoxon rank sum test, and the Wilcoxon signed‐rank test was used to compare paired data. Associations between continuous variables were assessed using Spearman's rank correlation (*ρ*). Two‐sided *P*‐values were calculated for all test statistics and *P* < 0.05 was considered significant. Data were visualised using the following R packages: ggpubr,^80^ cowplot,^81^ CATALYST.^27^

## Author contributions

**Stephen Tukwasibwe:** Conceptualization; formal analysis; investigation; methodology; project administration; writing – original draft; writing – review and editing. **Savannah Nicole Lewis:** Investigation; project administration; visualization; writing – original draft; writing – review and editing. **Yoweri Taremwa:** Data curation; investigation; writing – review and editing. **Kattria van der Ploeg:** Investigation; methodology; supervision; writing – review and editing. **Kathleen D Press:** Investigation; methodology; supervision; writing – review and editing. **Maureen Ty:** Methodology; supervision; writing – review and editing. **Felistas Namirimu Nankya:** Investigation; supervision; writing – review and editing. **Kenneth Musinguzi:** Investigation; writing – review and editing. **Evelyn Nansubuga:** Investigation; writing – review and editing. **Florian Bach:** Investigation; supervision; visualization; writing – review and editing. **Martin Chamai:** Investigation; writing – review and editing. **Martin Okitwi:** Investigation; writing – review and editing. **Gerald Tumusiime:** Investigation; writing – review and editing. **Annettee Nakimuli:** Supervision; writing – review and editing. **Francesco Colucci:** Supervision; writing – review and editing. **Moses R Kamya:** Funding acquisition; project administration; resources; writing – review and editing. **Joaniter I Nankabirwa:** Investigation; project administration; resources; writing – review and editing. **Emmanuel Arinaitwe:** Investigation; project administration; supervision; writing – review and editing. **Bryan Greenhouse:** Funding acquisition; project administration; supervision; writing − review and editing. **Grant Dorsey:** Funding acquisition; methodology; project administration; supervision; writing – review and editing. **Philip J Rosenthal:** Funding acquisition; project administration; supervision; writing – review and editing. **Isaac Ssewanyana:** Investigation; methodology; project administration; supervision; writing – review and editing. **Prasanna Jagannathan:** Conceptualization; data curation; formal analysis; funding acquisition; investigation; methodology; project administration; resources; supervision; validation; writing – review and editing.

## Conflict of interest

The authors declare no conflict of interest.

## Supporting information


Data S1

## Data Availability

The data that support the findings of this study are all included in this manuscript and supplementary figures. Any additional data or clarifications will be made available from the corresponding author upon reasonable request.

## References

[1] World malaria report 2023. Geneva: World Health Organization 2023. https://www.who.int/publications/i/item/9789240086173

[2] Doolan D , Dobaño C , Baird JK . Acquired immunity to malaria. Clin Microbiol Rev 2009; 22: 13–36. 19136431 10.1128/CMR.00025-08 PMC2620631

[3] Marsh K , Kinyanjui S . Immune effector mechanisms in malaria. Parasite Immunol 2006; 28: 51–60. 16438676 10.1111/j.1365-3024.2006.00808.x

[4] Langhorne J , Ndungu F , Sponaas AM , Marsh K . Immunity to malaria: more questions than answers. Nat Immunol 2008; 9: 725–732. 18563083 10.1038/ni.f.205

[5] Artavanis‐Tsakonas K , Tongren J , Riley E . The war between the malaria parasite and the immune system: immunity, immunoregulation, and immunopathology. Clin Exp Immunol 2003; 2: 145–152. 10.1046/j.1365-2249.2003.02174.x PMC1808775 12869017

[6] Portugal S , Pierce S , Crompton P . Young lives lost as B cells falter: what we are learning about antibody responses in malaria. J Immunol 2013; 190: 3039–3046. 23526829 10.4049/jimmunol.1203067 PMC3608210

[7] Crompton P , Kayala M , Traore B *et al*. A prospective analysis of the Ab response to *Plasmodium falciparum* before and after a malaria season by protein microarray. Proc Natl Acad Sci USA 2010; 107: 6958–6963. 20351286 10.1073/pnas.1001323107 PMC2872454

[8] Fowkes F , Richards J , Simpson J , Beeson J . The relationship between anti‐merozoite antibodies and incidence of *Plasmodium falciparum* malaria: a systematic review and meta‐analysis. PLoS Med 2010; 7: e1000218. 20098724 10.1371/journal.pmed.1000218 PMC2808214

[9] Feng G , Wines BD , Kurtovic L *et al*. Mechanisms and targets of Fcy‐receptor mediated immunity to malaria sporozoites. Nat Commun 2021; 12: 1742. 33741975 10.1038/s41467-021-21998-4 PMC7979888

[10] Chen Q , Amaladoss A , Ye W *et al*. Human natural killer cells control *Plasmodium falciparum* infection by eliminating infected red blood cells. Proc Natl Acad Sci USA 2014; 111: 1479–1484. 24474774 10.1073/pnas.1323318111 PMC3910619

[11] Arora G , Hart G , Manzella‐Lapeira J *et al*. NK cells inhibit *Plasmodium falciparum* growth in red blood cells via antibody‐dependent cellular cytotoxicity. elife 2018; 7: e36806. 29943728 10.7554/eLife.36806 PMC6019063

[12] Vivier E , Tomasello E , Baratin M , Walzer T , Ugolini S . Functions of natural killer cells. Nat Immunol 2008; 9: 503–510. 18425107 10.1038/ni1582

[13] Abel A , Yang C , Thakar M , Malarkannan S . Natural killer cells: development, maturation, and clinical utilization. Front Immunol 2018; 9: 1869. 30150991 10.3389/fimmu.2018.01869 PMC6099181

[14] Moebius J , Guha R , Peterson M *et al*. PD‐1 expression on NK cells in malaria‐exposed individuals is associated with diminished natural cytotoxicity and enhanced antibody‐dependent cellular cytotoxicity. Infect Immun 2020; 88: e00711‐19. 31907195 10.1128/IAI.00711-19 PMC7035929

[15] Hart G , Tran T , Theorel J *et al*. Adaptive NK cells in people exposed to *Plasmodium falciparum* correlate with protection from malaria. J Exp Med 2019; 216: 1280–1290. 30979790 10.1084/jem.20181681 PMC6547858

[16] Ty M , Sun S , Callaway P *et al*. Malaria‐driven expansion of adaptive‐like function CD56‐negative NK cells correlates with clinical immunity to malaria. Sci Transl Med 2023; 15: eadd9012. 36696483 10.1126/scitranslmed.add9012 PMC9976268

[17] Schlums H , Cichocki F , Tesi B *et al*. Cytomegalovirus infection drives adaptive epigenetic diversification of NK cells with altered signaling and effector function. Immunity 2015; 42: 443–456. 25786176 10.1016/j.immuni.2015.02.008 PMC4612277

[18] Lee J , Zhang T , Hwang I *et al*. Epigenetic modification and antibody‐dependent expansion of memory‐like NK cells in human cytomegalovirus‐infected individuals. Immunity 2015; 42: 431–442. 25786175 10.1016/j.immuni.2015.02.013 PMC4537797

[19] Tesi B , Schlums H , Cichocki F , Bryceson Y . Epigenetic regulation of adaptive NK cell diversification. Trends Immunol 2016; 37: 451–461. 27160662 10.1016/j.it.2016.04.006

[20] Larsen MD , Lopez‐Perez M , Dickson EK *et al*. Afucosylated *Plasmodium falciparum*‐specific IgG is induced by infection but not by subunit vaccination. Nat Commun 2021; 12: 5838. 34611164 10.1038/s41467-021-26118-w PMC8492741

[21] Odera D , Tuju J , Mwai K *et al*. Anti‐merozoite antibodies induce natural killer effector function and are associated with immunity against malaria. Sci Transl Med 2023; 15: eabn5993. 36753561 10.1126/scitranslmed.abn5993 PMC7616656

[22] Nankabirwa JI , Bousema T , Blanken SL *et al*. Measures of malaria transmission, infection, and disease in an area bordering two districts with and without sustained indoor residual spraying of insecticide in Uganda. PLoS One 2022; 17: e0279464. 36584122 10.1371/journal.pone.0279464 PMC9803187

[23] Alter G , Malenfant J , Altfeld M . CD107a as a functional marker for the identification of natural killer cell activity. J Immunol Methods 2004; 294: 15–22. 15604012 10.1016/j.jim.2004.08.008

[24] Farrington LA , Callaway PC , Vance HM *et al*. Opsonized antigen activates Vδ2+ T cells via CD16/FcγRIIIa in individuals with chronic malaria exposure. PLoS Pathog 2020; 16: e1008997. 33085728 10.1371/journal.ppat.1008997 PMC7605717

[25] Goodier M , Wolf AS , Riley E . Differentiation and adaptation of natural killer cells for anti‐malarial immunity. Immunol Rev 2020; 293: 25–37. 31762040 10.1111/imr.12798

[26] Cooper M , Fehniger T , Caligiuri M . The biology of human natural killer‐cell subsets. Trends Immunol 2001; 22: 633–640. 11698225 10.1016/s1471-4906(01)02060-9

[27] Chevrier S , Crowell H , Zanotelli V *et al*. Compensation of signal spillover in suspension and imaging mass cytometry. Cell Syst 2018; 6: 612–620. 29605184 10.1016/j.cels.2018.02.010 PMC5981006

[28] Moser K , Drábek E , Dwivedi A *et al*. Strains used in whole organism *Plasmodium falciparum* vaccine trials differ in genome structure, sequence, and immunogenic potential. Genome Med 2020; 12: 6. 31915075 10.1186/s13073-019-0708-9 PMC6950926

[29] Neafsey D , Juraska M , Bedford T *et al*. Genetic diversity and protective efficacy of the RTS,S/AS01 malaria vaccine. N Engl J Med 2015; 373: 2025–2037. 26488565 10.1056/NEJMoa1505819 PMC4762279

[30] Yeka A , Nankabirwa J , Mpimbaza A *et al*. Factors associated with malaria parasitemia, anemia and serological responses in a Spectrum of epidemiological settings in Uganda. PLoS One 2015; 10: e0118901. 25768015 10.1371/journal.pone.0118901 PMC4358889

[31] Ssewanyana I , Arinaitwe E , Nankabirwa JI *et al*. Avidity of anti‐malarial antibodies inversely related to transmission intensity at three sites in Uganda. Malar J 2017; 16: 67. 28183299 10.1186/s12936-017-1721-3 PMC5301436

[32] Ssewanyana I , Rek J , Rodriguez I *et al*. Impact of a rapid decline of malaria transmission on antimalarial IgG subclasses and avidity. Front Immunol 2021; 11: 576663. 33584643 10.3389/fimmu.2020.576663 PMC7873448

[33] Kamya MR , Arinaitwe E , Wanzira H *et al*. Malaria transmission, infection, and disease at three sites with varied transmission intensity in Uganda: implications for malaria control. Am J Trop Med Hyg 2015; 92: 903–912. 25778501 10.4269/ajtmh.14-0312 PMC4426576

[34] Gong L , Parikh S , Rosenthal PJ , Greenhouse B . Biochemical and immunological mechanisms by which sickle cell trait protects against malaria. Malar J 2013; 12: 317. 24025776 10.1186/1475-2875-12-317 PMC3847285

[35] Williams TN , Mwangi TW , Roberts DJ *et al*. An immune basis for malaria protection by the sickle cell trait. PLoS Med 2005; 2: e128. 15916466 10.1371/journal.pmed.0020128 PMC1140945

[36] Gong L , Maiteki‐Sebuguzi C , Rosenthal PJ *et al*. Evidence for both innate and acquired mechanisms of protection from *Plasmodium falciparum* in children with sickle cell trait. Blood 2012; 119: 3808–3814. 22327223 10.1182/blood-2011-08-371062 PMC3335384

[37] Zehner N , Adrama H , Kakuru A *et al*. Age‐related changes in malaria clinical phenotypes during infancy are modified by sickle cell trait. Clin Infect Dis 2021; 73: 1887–1895. 33738485 10.1093/cid/ciab245 PMC8599196

[38] Hwang I , Zhang T , Scott JM *et al*. Identification of human NK cells that are deficient for signaling adaptor FcRγ and specialized for antibody‐dependent immune functions. Int Immunol 2012; 24: 793–802. 22962434 10.1093/intimm/dxs080 PMC3621379

[39] Zhang T , Scott JM , Hwang I , Kim S . Cutting edge: antibody‐dependent memory‐like NK cells distinguished by FcRγ deficiency. J Immunol 2013; 190: 1402–1406. 23345329 10.4049/jimmunol.1203034 PMC3623944

[40] Cerwenka A , Lanier L . Natural killer cell memory in infection, inflammation, and cancer. Nat Rev Immunol 2016; 16: 112–123. 26806484 10.1038/nri.2015.9

[41] Paust S , Blish CA , Reeves RK . Redefining memory: building the case for adaptive NK cells. Virol J 2017; 91: e00169‐17. 10.1128/JVI.00169-17 PMC5625515 28794018

[42] Sherratt S , Patel A , Baker DA , Riley EM , Goodier MR . Differential IL‐18 dependence of canonical and adaptive NK cells for antibody dependent responses to *P. falciparum* . Front Immunol 2020; 11: 533. 32296438 10.3389/fimmu.2020.00533 PMC7137096

[43] Artavanis‐Tsakonas K , Eleme K , McQueen KL *et al*. Activation of a subset of human NK cells upon contact with *Plasmodium falciparum*‐infected erythrocytes. J Immunol 2003; 171: 5396–5405. 14607943 10.4049/jimmunol.171.10.5396

[44] Baratin M , Roetynck S , Nouvelle B *et al*. Dissection of the role of PfEMP1 and ICAM‐1 in the sensing of *Plasmodium falciparum*‐infected erythrocytes by natural killer cells. PLoS One 2007; 2: e228. 17311092 10.1371/journal.pone.0000228 PMC1794133

[45] Böttger E , Multhoff G , Kun JFJ , Esen M . *Plasmodium falciparum‐*infected erythrocytes induce granzyme B by NK cells through expression of host‐Hsp70. PLoS One 2012; 7: e33774. 22438997 10.1371/journal.pone.0033774 PMC3305334

[46] Saito F , Hirayasu K , Stash T *et al*. Immune evasion of *Plasmodium falciparum* by RIFIN via inhibitory receptors. Nature 2017; 552: 101–105. 29186116 10.1038/nature24994 PMC5748893

[47] Sakoguchi A , Arase H . Mechanisms for host immune evasion mediated by *Plasmodium falciparum*‐infected erythrocyte surface antigens. Front Immunol 2022; 13: 901864. 35784341 10.3389/fimmu.2022.901864 PMC9240312

[48] Björkström N , Ljunggren HG , Sandberg J . CD56 negative NK cells: origin, function, and role in chronic viral disease. Trends Immunol 2010; 31: 401–406. 20829113 10.1016/j.it.2010.08.003

[49] Cocker A , Guethlein L , Parham P . The CD56^−^ CD16^+^ NK cell subset in chronic infections. Biochem Soc Trans 2023; 51: 1201–1212. 37140380 10.1042/BST20221374

[50] Forconi C , Oduor C , Oluoch P *et al*. A new Hope for CD56^neg^ CD16^pos^ NK cells as unconventional cytotoxic mediators: an adaptation to chronic diseases. Front Cell Infect Microbiol 2020; 10: 162. 32373555 10.3389/fcimb.2020.00162 PMC7186373

[51] Rückert T , Lareau CA , Mashreghi MF , Ludwig LS , Romagnani C . Clonal expansion and epigenetic inheritance of long‐lasting NK cell memory. Nat Immunol 2023; 23: 1551–1563. 10.1038/s41590-022-01327-7 PMC9663309 36289449

[52] Chen Q , Schlichtherle M , Wahlgren M . Molecular aspects of severe malaria. Clin Microbiol Rev 2000; 13: 439–450. 10885986 10.1128/cmr.13.3.439-450.2000 PMC88942

[53] Deroost K , Pham TT , Opdenakker G , van den Steen P . The immunological balance between host and parasite in malaria. FEMS Microbiol Rev 2016; 40: 208–257. 26657789 10.1093/femsre/fuv046

[54] Frimpong A , Amponsah J , Adjokatseh AS *et al*. Asymptomatic malaria infection is maintained by a balanced pro‐ and anti‐inflammatory response. Front Microbiol 2020; 11: 559255. 33281757 10.3389/fmicb.2020.559255 PMC7705202

[55] Luetke‐Eversloh M , Hammer Q , Durek P *et al*. Human cytomegalovirus drives epigenetic imprinting of the *IFNG* locus in NKG2C^hi^ natural killer cells. PLoS Pathog 2014; 10: e1004441. 25329659 10.1371/journal.ppat.1004441 PMC4199780

[56] Epstein JE , Paolino KM , Richie TL *et al*. Protection against *Plasmodium falciparum* malaria by PfSPZ vaccine. JCI Insight 2017; 2: e89154. 28097230 10.1172/jci.insight.89154 PMC5214067

[57] Lyke KE , Ishizuka AS , Berry AA *et al*. Attenuated PfSPZ vaccine induces strain‐transcending T cells and durable protection against heterologous controlled human malaria infection. Proc Natl Acad Sci USA 2017; 114: 2711–2716. 28223498 10.1073/pnas.1615324114 PMC5347610

[58] Kyes SA , Kramer SM , Smith JD . Antigenic variation in *Plasmodium falciparum*: gene organization and regulation of the var multigene family. Eukaryot Cell 2007; 6: 1511–1520. 17644655 10.1128/EC.00173-07 PMC2043368

[59] Peters JM , Fowler EV , Krause DR , Cheng Q , Gatton ML . Differential changes in *Plasmodium falciparum var* transcription during adaptation to culture. J Infect Dis 2007; 195: 748–755. 17262719 10.1086/511436 PMC1866257

[60] Quadt KA , Barfod L , Andersen D *et al*. The density of knobs on *Plasmodium falciparum‐*infected erythrocytes depends on developmental age and varies among isolates. PLoS One 2012; 7: e45658. 23029166 10.1371/journal.pone.0045658 PMC3447797

[61] Zhang Q , Zhang Y , Huang Y *et al*. From *in vivo* to *in vitro*: dynamic analysis of *Plasmodium falciparum* var gene expression patterns of patient isolates during adaptation to culture. PLoS One 2011; 6: e20591. 21674009 10.1371/journal.pone.0020591 PMC3108956

[62] Tilly AK , Thiede J , Metwally N *et al*. Type of *in vitro* cultivation influences cytoadhesion, knob structure, protein localization and transcriptome profile of *Plasmodium falciparum* . Sci Rep 2015; 5: 16766. 26568166 10.1038/srep16766 PMC4645185

[63] Frankland S , Elliott SR , Yosaatmadja F *et al*. Serum lipoproteins promote efficient presentation of the malaria virulence protein PfEMP1 at the erythrocyte surface. Eukaryot Cell 2007; 6: 1584–1594. 17644656 10.1128/EC.00063-07 PMC2043375

[64] Ibison F , Olotu A , Muema DM *et al*. Lack of avidity maturation of merozoite antigen‐specific antibodies with increasing exposure to *Plasmodium falciparum* amongst children and adults exposed to endemic malaria in Kenya. PLoS One 2012; 7: e52939. 23300828 10.1371/journal.pone.0052939 PMC3530478

[65] Chiu CYH , Healer J , Thompson JK *et al*. Association of antibodies to *Plasmodium falciparum* reticulocyte binding protein homolog 5 with protection from clinical malaria. Front Microbiol 2014; 5: 314. 25071730 10.3389/fmicb.2014.00314 PMC4074990

[66] Weaver R , Reiling L , Feng G *et al*. The association between naturally aquired IgG subclass specific antibodies to the PfRH5 invasion complex and protection from *Plasmodium falciparum* malaria. Sci Rep 2016; 6: 33094. 27604417 10.1038/srep33094 PMC5015043

[67] Roussillon C , Oeuvray C , Müller‐Graf C *et al*. Long‐term clinical protection from *falciparum* malaria is strongly associated with IgG3 antibodies to merozoite surface protein 3. PLoS Med 2007; 4: e320. 18001147 10.1371/journal.pmed.0040320 PMC2071934

[68] Stanisic DI , Richards JS , McCallum FJ *et al*. Immunoglobulin G subclass‐specific responses against *Plasmodium falciparum* merozoite antigens are associated with control of parasitemia and protection from symptomatic illness. Infect Immun 2009; 77: 1165–1174. 19139189 10.1128/IAI.01129-08 PMC2643653

[69] Tessema SK , Nakajima R , Jasinskas A *et al*. Protective immunity against severe malaria in children is associated with a limited repertoire of antibodies to conserved PfEMP1 variants. Cell Host Microbe 2019; 26: 579–590. 31726028 10.1016/j.chom.2019.10.012

[70] Garraud O , Mahanty S , Perrault R . Malaria‐specific antibody subclasses in immune individuals: a key source of information for vaccine design. Trends Immunol 2003; 24: 30–35. 12495722 10.1016/s1471-4906(02)00012-1

[71] Hviid L , Lopez‐Perez M , Larsen MD , Vidarsson G . No sweet deal: the antibody‐mediated immune response to malaria. Trends Parasitol 2022; 38: 428–434. 35279381 10.1016/j.pt.2022.02.008

[72] Lopez‐Perez M , Seidu Z , Larsen M *et al*. Acquisition of fc‐afucosylation of PfEMP1‐specific IgG is age‐dependent and associated with clinical protection against malaria. Res Sq 2024. doi:10.21203/rs.3.rs-4165378/v1 PMC11696684 39747065

[73] Cholera R , Brittain NJ , Gillrie MR *et al*. Impaired cytoadherence of *Plasmodium falciparum*‐infected erythrocytes containing sickle hemoglobin. Proc Natl Acad Sci USA 2008; 105: 991–996. 18192399 10.1073/pnas.0711401105 PMC2242681

[74] Simoni Y , Fehlings M , Kløverpris HN *et al*. Human innate lymphoid cell subsets possess tissue‐type based heterogeneity in phenotype and frequency. Immunity 2017; 46: 148–161. 27986455 10.1016/j.immuni.2016.11.005 PMC7612935

[75] Piriou E , Asito AS , Sumba PO *et al*. Early age at time of primary Epstein‐Barr virus infection results in poorly controlled viral infection in infants from Western Kenya: clues to the etiology of endemic Burkitt lymphoma. J Infect Dis 2012; 205: 906–913. 22301635 10.1093/infdis/jir872 PMC3282570

[76] Kurtovic L , Atre T , Feng G *et al*. Multifunctional antibodies are induced by the RTS,S malaria vaccine and associated with protection in a phase 1/2a trial. J Infect Dis 2021; 224: 1128–1138. 32236404 10.1093/infdis/jiaa144 PMC8514181

[77] Beeson JG , Kurtovic L , Dobaño C *et al*. Challenges and strategies for developing efficacious and long‐lasting malaria vaccines. Sci Transl Med 2019; 11: eaau1458. 30626712 10.1126/scitranslmed.aau1458

[78] Hahne F , LeMeur N , Brinkman RR *et al*. flowCore: a Bioconductor package for high throughput flow cytometry. BMC Bioinform 2009; 10: 106. 10.1186/1471-2105-10-106 PMC2684747 19358741

[79] Weber LM , Nowicka M , Soneson C , Robinson MD . Diffcyt: differential discovery in high‐dimensional cytometry via high‐resolution clustering. Commun Bio 2019; 2: 183. 31098416 10.1038/s42003-019-0415-5 PMC6517415

[80] Kassambara A . ggpubr: ‘ggplot2’ Based Publication Ready Plots. R package version 0.6.0. 2023. https://rpkgs.datanovia.com/ggpubr/.

[81] Wilke C . cowplot: streamlined Plot Theme and Plot Annotations for ‘ggplot2’. R package version 1.1.3. 2024. https://wilkelab.org/cowplot/

