# Supplementary material for: Natural killer cell antibody‐dependent cellular cytotoxicity to Plasmodium falciparum is impacted by cellular phenotypes, erythrocyte polymorphisms, parasite diversity and intensity of transmission
Source: Clin Transl Immunology. 2024 Nov 1;13(11):e70005. doi: 10.1002/cti2.70005 (PMC11528551; doi:10.1002/cti2.70005)
Supplement: Supplementary file 1 — Data S1 [file CTI2-13-e70005-s001.pdf]

Supporting information

Tukwasibwe & Lewis, *et al*. 2024

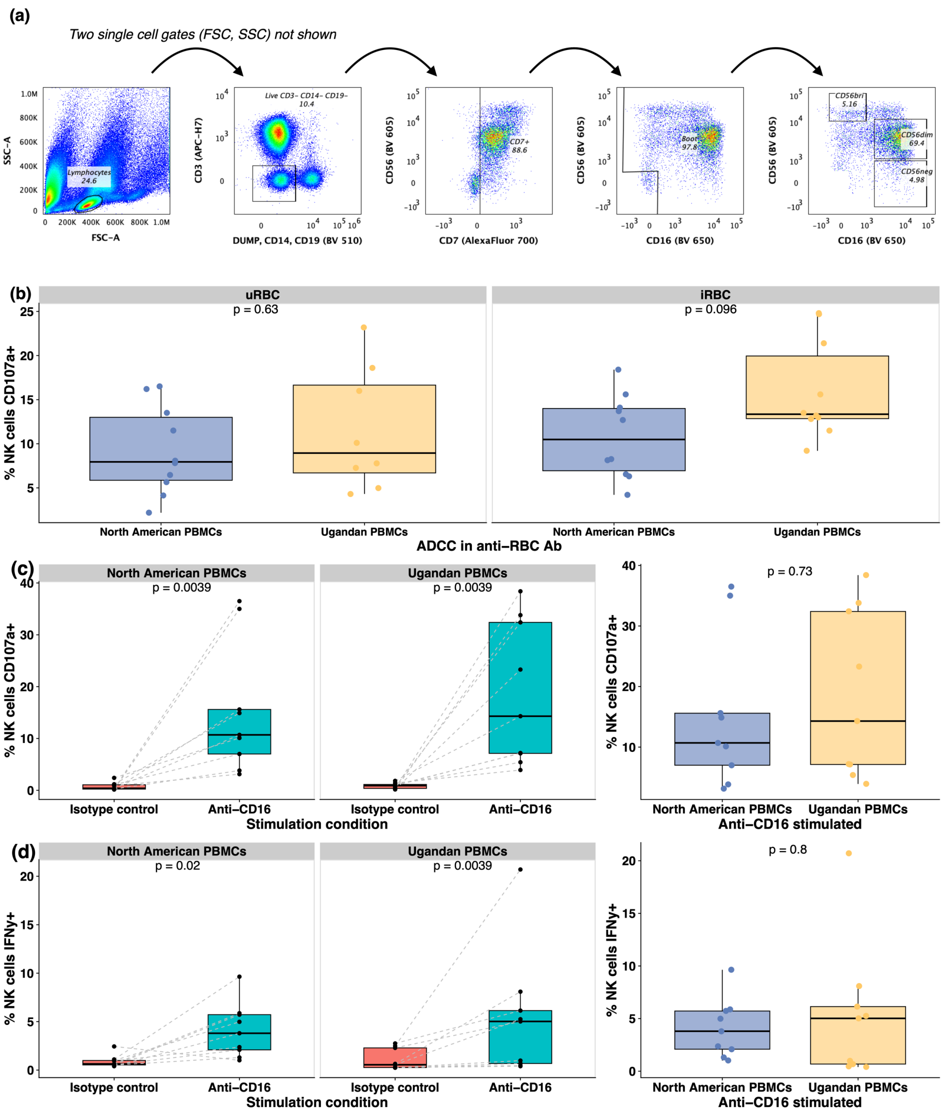

<u>Supplementary figure 1</u>: **(a)** Flow cytometry gating strategy used to filter NK cells from PBMCs (left to right). First, a tight lymphocyte gate was drawn. After two additional gates were drawn to exclude doublets, one using forward scatter and one using side scatter (not shown), live, CD3-, CD14-, and CD19- cells were gated on for further analysis. From that population, we gated on CD7+ cells. Using the markers CD56 and CD16, the double negative population was removed from further analysis (excluded from the "Boot" gate), and NK cell subpopulations are gated on as shown in the final rightmost panel. **(b)** Comparison of NK cell degranulation in response to uRBCs (left panel) and iRBCs (right panel) opsonized in a polyclonal anti-RBC antibody from the experiments in **Figure 1** (n = 10 North American and Ugandan PBMC donors). P-values were calculated using Wilcoxon rank sum tests. Plots quantifying and comparing CD107a **(c)** and IFNɣ (d) production by NK cells (North American and Ugandan PBMC donors, n = 9 each) in a plate-bound CD16 crosslinking assay. *P*-values were calculated using Wilcoxon rank sum tests: the tests were paired in the leftmost panels (same donors in different conditions) and unpaired on the right (different donors).

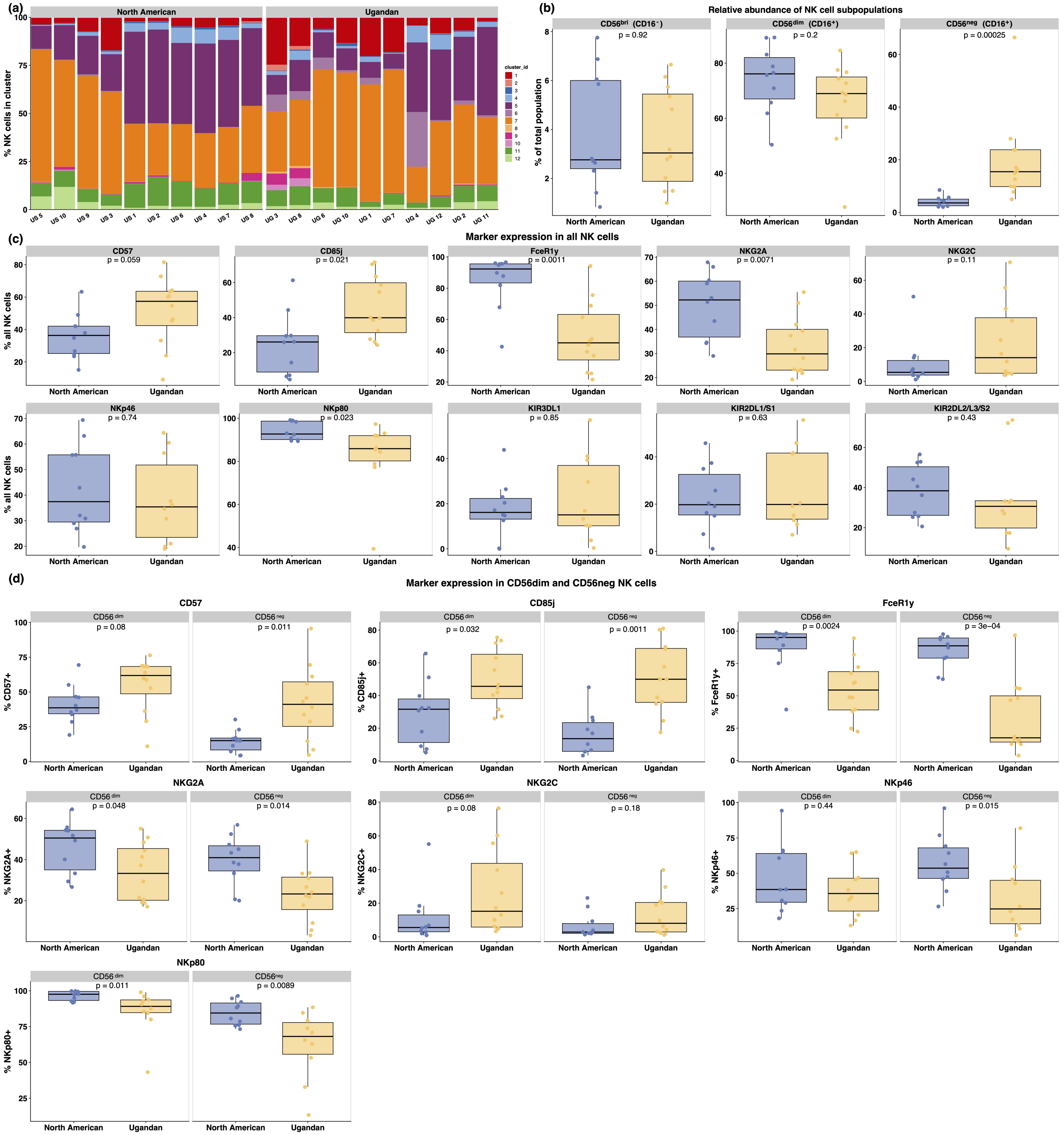

<u>Supplementary figure 2</u>: **(a)** Stacked bar plots representing the individual-level abundance of NK cell clusters from unsupervised analyses included in **Figure 3**. The data that follow are manual (supervised) gating analyses of NK cell phenotypes between North American (n = 10) and Ugandan (n = 12) PBMC donors. **(b)** Relative abundance of NK cell subpopulations. **(c)** Population-wide expression of individual markers. **(d)** Expression of individual markers in CD56dim and CD56neg NK cell populations. *P*-values were calculated using Wilcoxon rank sum tests.

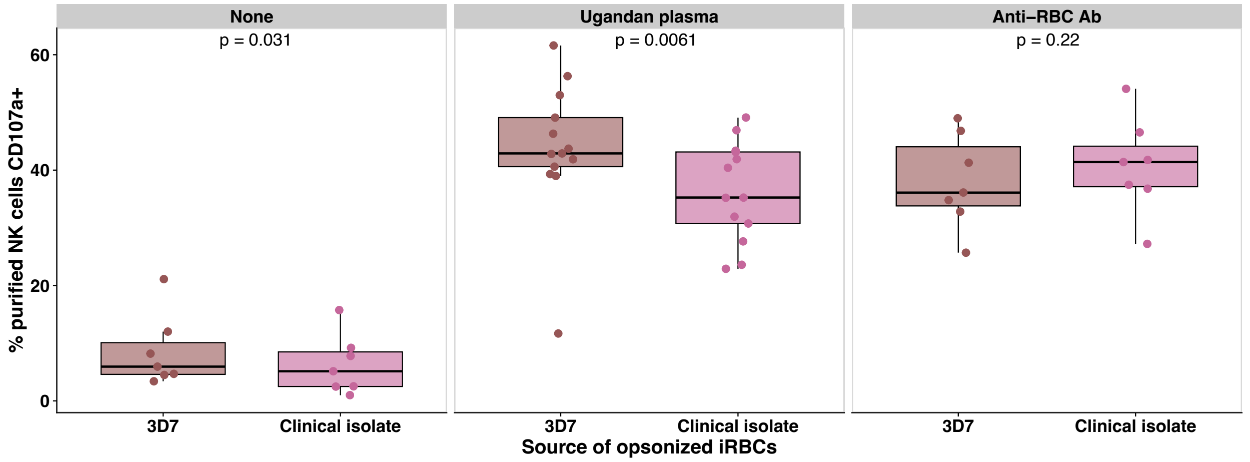

<u>Supplementary figure 3</u>: NK cell degranulation was lower in response to opsonized clinical *Pf* isolates compared to the 3D7 lab strain. Schizont-stage iRBCs were obtained from cultured 3D7 and clinical isolate *Pf* (n = 6 isolates). NK cells purified from adult Ugandan PBMC donors (n = 6) were stimulated with iRBCs alone or iRBCs opsonized in pooled Ugandan plasma or an anti-RBC antibody. *P*-values were calculated using paired Wilcoxon rank sum tests.

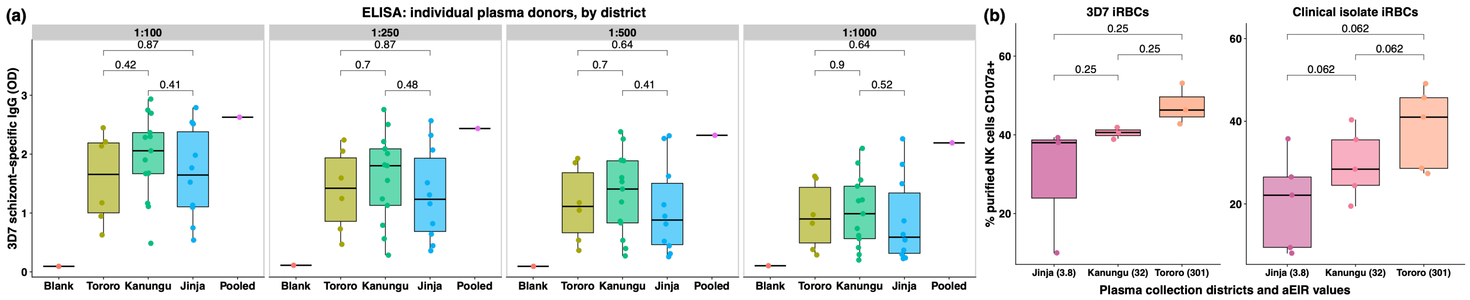

Supplementary figure 4: **(a)** Enzyme-linked immunosorbent assay (ELISA) quantifying IgG titers specific to 3D7 schizont lysate in the individual plasma donors (n = 6 [Tororo], n = 13 [Kanungu], n = 10 [Jinja]) used to create plasma pools used in **Figure 5**, as measured by optical density (OD). **(b)** Subset of **Figure 5a** data showing degranulation in response to 3D7 iRBCs (left panel) and clinical isolate iRBCs (right panel). *P*-values were calculated using paired Wilcoxon rank sum tests.

Supplementary table 1

Characteristics of Ugandan PBMC donors (as related to Figure 1-2)

	**Study cohort** (N = 15)
Enrolled in PRISM Border Cohort study, n/N (%)	13/15 (86.7%)
Donated blood at Mbale Regional Referral Hospital, n/N (%)	2/15 (13.3%)

	**PRISM Border Cohort** (N = 13)
Age in years at time of sampling, mean (SD)	35.8 (10.7)
Male sex, n/N (%)	5/13 (38.5%)
Parasitemia at time of sampling by blood smear, n/N (%)	4/13 (30.8%)
Parasitemia at time of sampling by qPCR, n/N (%)	5/13 (38.5%)

Supplementary table 2

Characteristics of Ugandan plasma donors (as related to Figure 4)			
	**PRISM 1 Study site**		
Location (aEIR)	Jinja (3.8)	Kanungu (32)	Tororo (301)
Residents, n/N (%)	10/27 (37)	11/27 (40.7)	6/27 (22.2)
Male sex, n/N (%)	5/10 (50)	4/11 (36.4)	2/6 (33.3)
Age in years at time of sampling, mean (SD)	8.03 (1.04)	8.16 (1.18)	8.84 (0.791)
Parasitemia at time of sampling by blood smear, n/N (%)	0/10	0/11	0/6
Parasitemia at time of sampling by blood smear, n/N (%)	0/10	0/11	0/6
